# Supplementary material for: Enrichment of root-associated Streptomyces strains in response to drought is driven by diverse functional traits and does not predict beneficial effects on plant growth
Source: PLoS Biol. 2025 Nov 26;23(11):e3003526. doi: 10.1371/journal.pbio.3003526 (PMC12698021; doi:10.1371/journal.pbio.3003526)
Supplement: Attachment — Submitted filename: Response to Reviewers comments.docx [file pbio.3003526.s009.docx]

Dear Dr Coleman-Derr,

Thank you for your patience while your manuscript "Uncovering the hidden diversity and functional roles of root endophytic Streptomyces under drought stress" was peer-reviewed at PLOS Biology. It has now been evaluated by the PLOS Biology editors, an Academic Editor with relevant expertise, and by three independent reviewers. Please accept my sincere apologies for the delay.

In light of the reviews, which you will find at the end of this email, we would like to invite you to revise the work to thoroughly address the reviewers' reports. As you will see below, majority of reviewers are positive about the relevance and novelty of the study, yet some concerns have raised during revision. Reviewer 1 has several questions like the differences in taxonomic composition (which may require validation) and if analysing the linkage between exametabolomic molecules and DE/PGP scores might be more direct evidence. Reviewer 2 has several comments on how the study is written and organized. Reviewer 3 suggests to expand the analysis on gene neighbourhoods with a focus on FabG, as well as on the impact of monoassociation on the plant. **We agree with all reviewer concerns and would require some additional experimental revisions to address them, as we consider that this would strengthen the work.**

IMPORTANT: after discussion with the Academic Editor and the reviewers, we would like to **emphasize point 2 of reviewer 3** as this will give a focus on the plant physiological responses to drought.

Given the extent of revision needed, we cannot make a decision about publication until we have seen the revised manuscript and your response to the reviewers' comments. Your revised manuscript is likely to be sent for further evaluation by all or a subset of the reviewers.

In addition to these revisions, you will need to complete some formatting changes, which you will receive in a follow up email. A member of our team will be in touch with a set of requests shortly.

We expect to receive your revised manuscript within 3 months. Please email us (plosbiology@plos.org) if you have any questions or concerns, or would like to request an extension.

At this stage, your manuscript remains formally under active consideration at our journal; please notify us by email if you do not intend to submit a revision so that we may withdraw it.

**IMPORTANT - SUBMITTING YOUR REVISION**

Your revisions should address the specific points made by each reviewer. Please submit the following files along with your revised manuscript:

1. A 'Response to Reviewers' file - this should detail your responses to the editorial requests, present a point-by-point response to all of the reviewers' comments, and indicate the changes made to the manuscript.

*NOTE: In your point-by-point response to the reviewers, please provide the full context of each review. Do not selectively quote paragraphs or sentences to reply to. The entire set of reviewer comments should be present in full and each specific point should be responded to individually, point by point.

You should also cite any additional relevant literature that has been published since the original submission and mention any additional citations in your response.

2. In addition to a clean copy of the manuscript, please also upload a 'track-changes' version of your manuscript that specifies the edits made. This should be uploaded as a "Revised Article with Changes Highlighted" file type.

*Re-submission Checklist*

When you are ready to resubmit your revised manuscript, please refer to this re-submission checklist: <https://gcc02.safelinks.protection.outlook.com/?url=https%3A%2F%2Fplos.io%2FBiology_Checklist&data=05%7C02%7Cdevin.coleman-derr%40usda.gov%7Cca73a98e21634cfd743b08ddea51e0ae%7Ced5b36e701ee4ebc867ee03cfa0d4697%7C1%7C0%7C638924359576069543%7CUnknown%7CTWFpbGZsb3d8eyJFbXB0eU1hcGkiOnRydWUsIlYiOiIwLjAuMDAwMCIsIlAiOiJXaW4zMiIsIkFOIjoiTWFpbCIsIldUIjoyfQ%3D%3D%7C0%7C%7C%7C&sdata=WEJmrA3cRhWJUnjvyfzhX11LXfX9WpZ0dBfK6BKXaxA%3D&reserved=0>

To submit a revised version of your manuscript, please go to <https://gcc02.safelinks.protection.outlook.com/?url=https%3A%2F%2Fwww.editorialmanager.com%2Fpbiology%2F&data=05%7C02%7Cdevin.coleman-derr%40usda.gov%7Cca73a98e21634cfd743b08ddea51e0ae%7Ced5b36e701ee4ebc867ee03cfa0d4697%7C1%7C0%7C638924359576090076%7CUnknown%7CTWFpbGZsb3d8eyJFbXB0eU1hcGkiOnRydWUsIlYiOiIwLjAuMDAwMCIsIlAiOiJXaW4zMiIsIkFOIjoiTWFpbCIsIldUIjoyfQ%3D%3D%7C0%7C%7C%7C&sdata=V6z4SJAOB8w6A%2BHzVSdMr9EDi4PcS4%2FsUOvqf6SYp7g%3D&reserved=0> and log in as an Author. Click the link labelled 'Submissions Needing Revision' where you will find your submission record.

Please make sure to read the following important policies and guidelines while preparing your revision:

*

Thank you again for your submission to our journal. We hope that our editorial process has been constructive thus far, and we welcome your feedback at any time. Please don't hesitate to contact us if you have any questions or comments.

Sincerely,

Melissa

Melissa Vazquez Hernandez, Ph.D.

Associate Editor

PLOS Biology

mvazquezhernandez@plos.org

------------------------------------

REVIEWERS' COMMENTS

------------------------------------

**Reviewer #1 (Yi Song):**

Drought is one of the most serious abiotic stresses for agricultural production worldwide, while we have very limited biotechnologies to combat drought relative to pathogens. Accumulating evidence suggest that plant microbiome changes contribute to plant drought tolerance, however, the microbial ecologic and genetic mechanisms underlying drought induced microbiome changes are still not clear. The authors tried to conduct in-depth mechanistic exploration for their previous found interesting phenomenon: the genus Streptomyces is consistently enriched in the roots of drought-stressed plants and confer drought protecting activity for crops. One of the really cool point of this manuscript is that they carefully combined sequencing based microbiome analyses and isolates based strain functional and genomic analyses. This basically is a perfect "binning" process similar to metagenomics based microbe genome assembly, but definitely much higher accuracy because they successfully

isolated and purified representative strains. They further explored the physiological and genomic correlations with their drought enrichment folds, and found that there is not strong correlation between drought enrichment folds and potential drought protection ability. This is of significance to guide the characterization and application of beneficial microbiome in agriculture. Overall the MS is rigorously designed and clearly written.

*We thank the reviewer for their positive assessment of the manuscript. We have attempted to address the specific points raised below.*

I have a couple questions related to the manuscript:

[1] One of my biggest surprise is that there are huge differences in the taxonomic composition between full-length and V3-V4 region based 16S rRNA gene sequencing results (1% Actinobacteria in full-length control group, while around 30% in v3-v4 approach?). This part lacks potential explanation or cross validations? Are there any similar comparison study in animal gut or environmental systems? Is this common to observe such a drastic (or totally different) differences between those two approaches?

*It is quite common to see significant differences in taxonomic representation in 16S rRNA datasets generated with different primer sets. We have included references to papers that highlight that this is to be expected, and additionally we have added a new supplemental table (S2 Table) that reports an* in silico *predicted primer coverage of four of the most dominant phyla in our dataset (including the Actinobacteria). This data was generated using the Silva SSU Ref NR database (release 132); it is reported for both the full length PacBio primers (27F-1492R) as well as the V3-V4 Illumina primers (341F-785R), and includes both the predicted (in silico) and observed results. From these data, it is clear that Actinobacteria are less well covered by the full length primers used than the other three phyla. Importantly, none of our downstream results or components central to the main message hinge on seeing similar levels of amplification of Actinobacterial sequences across the two platforms, and the key point of a differential abundance of Actinobacteria under drought and watered conditions is observable in both datasets.*

*[Line 148, Track Changes Doc]*

*“Interestingly, Actinobacterial amplification was lower in both conditions when the full-length 16S rRNA primers were used (****S1 Table****). An* in silico *analysis of Actinobacteria, Proteobacteria, Bacteroidetes, and Firmicutes sequences in the reference database used for taxonomic classification revealed that a much smaller fraction of Actinobacterial sequences were ‘amplifiable’ with the PacBio full-length primer set than with the V3-V4 primer set, potentially accounting for the discrepancy, and numerous studies have demonstrated that use of different 16S rRNA primer sets can lead to significant and reproducible differences in the resulting taxonomic profiles* [*(Abellan-Schneyder et al. 2021; Tremblay et al. 2015; Waechter et al. 2023)*](https://paperpile.com/c/VV6dlk/21bA+fGcB+mZq0)*. Importantly, however, both the V3-V4 and the full-length 16S datasets demonstrate that Actinobacteria are significantly enriched in the roots of drought-stressed sorghum.“*

[2] I would be a bit more careful to draw the conclusion that "this challenges the conventional 'cry for help' framework." Because the "cry for help" concept itself is not a strict "mathematical law", in contrast, it is more like an "ecological trend" which usually helpful to guide the predication and discovery of potential beneficial microbes at the family or genus level. It helps researchers to narrow down potential "target" microbes for functional validations. Moreover, the "cry for help" phenomenon seems to be supported by a lot genetic and metabolic evidence from host side. Meanwhile, the input from this study (DE) was got from mono-association system and Quantitative profiling, which is very different from the relative abundance and natural soil community (which was usually the input for "cry for help" studies). Recent study also indicate the importance and relevance for relative fold changes for predicting core drought responsive microbes [ PMC12043206]. I guess simply delete line 439-441 could address this issue.

*[Line 527, Track Changes Doc]*

*We appreciate the Reviewer’s point and agree that the suggested change would benefit the manuscript as a whole. We have followed the advice to delete the suggested lines.*

[3] The authors successfully isolated diverse Streptomyces strains and performed exometabolomic profiling studies, which is cool. However, the authors only analyzed the correlation between genomic loci and DE scores or growth promoting effect in the study. I am wondering would that be more direct to analyze the linkage between exometabolomic molecules and DE/PGP scores? Would that be more direct to reveal potential rules of small molecules controlling positive DE scores in Streptomyces strains.

*This is an interesting suggestion and one we had considered. Unfortunately, while we have measured DE score and growth promotion in our fully sequenced cohort of 48 Streptomyces, we only have exometabolomics data for the much smaller subset of 12 DC strains. We have attempted the suggested analysis to correlate DE with various phenotypes among the 12 DC strains. Unfortunately, we found that in all cases, a sample size of 12 isolates was insufficient to yield significant linkage between DE/PGP scores and metabolomic profiles.*

[4] fig3 seems hard to understand. Is it lacking scale bar? Is there an easier way to illustrate this, or could we separate them into more clear different graphs?

*[Line 265, Track Changes Doc]*

*We appreciate the Reviewer’s point that, as originally produced, Figure 3 is challenging to interpret. We have made modifications to the legend and the figure to help improve the interpretability of the figure, including relabelling the figure with clearer identifiers and larger text.*

------------------------------------

**Reviewer #2:**

This manuscript presents an observational study on the genetic diversity of in bacterial family of Streptomyces often associated with plants under drought stress. The study uses well-established genomics methods to analyze diversity in the Streptomyces strains present in sorghum roots under drought stress. The results are not very surprising in that it is well known that related microbial strains can have vasty different functionality. The novelty of the study seems to be in linking drought enrichment with possible growth promotion and showing that there is no link. The manuscript, while relatively well written in the technical sense is hard to follow.

*We appreciate the Reviewer’s suggestion to modify the flow of the manuscript to improve its ease of interpretability. We have modified several sections of the manuscript to attempt to more clearly highlight what we find to be the most important message of the study. This has proved a highly valuable suggestion and we appreciate the Reviewer’s extensive comments below that helped us identify the sections that needed to be addressed.*

The results are presented as a mixture of hypotheses and methods and it remains unclear whether the main outcome of this study was the comparison between the two sequencing methods or the observations of the variability in functionality of the strains.

*[Lines 70-120, Track Changes Doc]*

*Our central purpose was to highlight the variability in functionality of the strains, but a secondary purpose was to expose the limitations of 16S rRNA sequencing (whether variable-region or full-length) as a sole means of inquiry. The direct comparison of variable region and full-length 16S sequencing should be seen as the first step on our path towards a deeper story, and we agree that an undo focus in the introduction may have set the stage for an expectation on the reader’s part that this would feature more prominently in the remainder of the manuscript. We have revised the introduction to reduce the emphasis on this section.*

The abstract and introduction emphasize the latter while the methods and

discussion emphasize the former. The emphasis on the sequencing techniques comes as a surprise to the reader because this aspect of the study is not mentioned in the introduction at all.

*We agree that the Introduction could benefit from a rework, with the removal of material on 16S rRNA amplicon sequencing techniques, which gives the impression that this will be a central focus of the study.*

*[Line 20, Track Changes Doc] The Abstract currently contains only a single statement regarding the use of comparative 16S rRNA profiling, and serves as the basis for introducing the topic of hidden complexity, whose accessibility is dependent on the platform used.*

*[Line 422-455, Track Changes Doc] The Discussion currently has only a single paragraph (out of five) exploring and discussing the 16S rRNA results from our study. We intended this paragraph to be primarily centered around the idea that it is best to pair 16S rRNA sequencing with other techniques if the goal is characterizing functional diversity within a microbiome, rather than a discussion of the two 16S platforms employed in the study.*

*[Line 616-652, Track Changes Doc] In the Methods section, we find only two of the ten sections describing work related to the 16S rRNA analysis, with the remainder of the sections devoted to field experiment design, microbial isolation, microbox experimental design, drought phenotyping, metabolomics, microbial phenotyping, and pangenome analysis.*

I suggest reorganizing the manuscript around one focus area (the one that the authors think has the highest impact/importance to the community) and clearly introducing the background of that including information about hypotheses and the methods to test the hypotheses.

*We have revised nearly all portions of the manuscript to attempt to address this, including a considerable rework of the Introduction [Line 70-120, Track Changes Doc], removing material associated with the background of 16S rRNA sequencing, and modifying the Discussion [Line 522-545, Track Changes Doc]. Ultimately, we believe these changes address this issue and improve the manuscript as a whole.*

In its current form, the introduction seems somewhat superficial about what is known about the genus Streptomyces.

*[Line 102-120, Track Changes Doc] We have added additional detail into the Introduction to expand the focus on Streptomyces functional diversity and its potential roles in the root microbiome.*

The discussion also revolves around methodology rather than the deeper meaning of the results. If the study focus is the diversity in functionality of Streptomyces strains, then discussing that and its implications for plant or microbiome performance would strengthen the manuscript.

*[Line 522-545, Track Changes Doc] We have expanded the final paragraph of the Discussion to include a greater focus on the potential implications of Streptomyces diversity in the rhizosphere for plant health, specifically the need for strain-level functional profiling in designing microbial-based products for use in agriculture.*

Specific comments:

-Terminology: Please define DE clearly in the manuscript and indicate when the text refers to the calculated DE value and when the word "enrichment" is used to refer to presence or absence of Streptomyces in the roots. Now these two terms are somewhat mixed making the manuscript hard to follow.

*We appreciate this suggestion; we have gone through each instance of the usage of DE within the text, as well as the first instance, and made an effort to be consistent and clear throughout the manuscript.*

*[Line 139, Track Changes Doc] “*The genus *Streptomyces* has repeatedly exhibited strong increased relative abundance under drought, relative to irrigated conditions (referred to as drought enrichment or DE) through a variety of metagenomics sequencing techniques [[5,8–10]](https://paperpile.com/c/5tZgH3/EnmQ+z2gA+oUO7+amEd), but the degree to which this DE holds for individual root-associated *Streptomyces* remains unclear.”

-Tenses: Please use the same tense consistently to present what was done in this study. Most journal like the style of everything done in this study presented using past tense. Now the methods and results jump between past and present tense.

*We have made the suggested change to use the past tense throughout the manuscript.*

Figures: Why is the phylogenic tree needed in Fig. 3? It is partially covered. If it is important, why is it not shown fully?

*We agree that the tree is not needed, and Figure 3 has been revised to fit with this suggestion. The original intent was to show that strains in two of the AC groups (AC6 and AC8) are in some cases more closely related to strains from a different AC group than their own AC group when considering whole genome ANI, but this point is made in the text and the legend.*

Also the results refer quite extensively to the figures in the supplementary materials. Please consider bringing the key figures that show results to the main text as this journal does not have a figure limitation.

*[Line 303-311, Track Changes Doc] This is an excellent suggestion; we have moved one of what we consider the more important Supplemental Figures (Figure G) to main figures (now Figure 4), and renumbered all figures accordingly.*

-Line 49: This seems like an overstatement as this study addresses only a limited number of strains in the genus.

*We have revised this statement to more clearly state that DE score did not predict Streptomyces isolates ability to promote plant growth in our specific subset of tested isolates.*

*[Line 49, Track Changes Doc] “Importantly, DE did not predict a Streptomyces’ ability to promote plant growth under stress in our collection of tested isolates.”*

-Line 77: It is unclear what "this response" refers to here. Please clarify.

*[Line 102-120, Track Changes Doc] We have revised this entire section to expand the focus on Streptomyces functional diversity and its potential roles in the root microbiome as requested by an earlier comment (see above).*

-Line 81-83: If the focus of the study is revealing functional diversity, please explain what aspect of that these methods can reveal.

*In this study, the central message is that it is the combined use of high-resolution, full-length 16S rRNA sequencing, isolate sequencing, metabolomics, and microbial phenotyping (or related suites of omic analysis) that enable a deeper understanding of functional diversity within complex microbiomes. We attempt to illustrate through our experiments that 16S rRNA sequencing, of any type, by itself is insufficient to capture the considerable strain- and isolate-level variation in key traits; in our case, this includes DE and plant growth promotion, among others.*

*We agree with the reviewer’s comment and have amended the referenced statements in the manuscript to more clearly reflect our work’s emphasis on the importance of functional diversity not observable by 16S rRNA sequencing alone:*

*[Line 102-120, Track Changes Doc] “While Streptomyces enrichment under drought is well-documented, less is known about the functional diversity within this genus that may explain its ecological success in the rhizosphere and its variable effects on plant hosts. Streptomyces species are renowned for their metabolic versatility, including the production of antibiotics, siderophores, osmoprotectants, and plant hormones, but the presence and expression of these traits can vary dramatically even among closely related strains* [*(Doroghazi et al. 2014; Viaene et al. 2016; Chandra and Chater )*](https://paperpile.com/c/VV6dlk/HHey+BsP9+uaHb)*. In drought-stressed rhizospheres, these functional differences could influence microbial fitness, competitive interactions, and ultimately, the degree of benefit conferred to the plant. We hypothesized that the drought enrichment (DE) of Streptomyces in sorghum roots is not a uniform, lineage-level phenomenon, but is instead driven by isolate-specific traits that vary across a genetically diverse pool of endophytic strains. Furthermore, we proposed that DE and its underlying causes – such as osmotic stress tolerance, secondary metabolite production, siderophore production, and the ability to utilize drought-associated root exudates—could be uncoupled from the strains' capacity to promote plant growth.*

-Line 104-107: Here the use of the word "enrichment" is confusing. Please clarify what is the metric of enrichment here.

*[Line 139, Track Changes Doc] We have revised this statement to increase readability. It is now:*

*“*The genus *Streptomyces* has been repeatedly found to exhibit strong increased relative abundance under drought, relative to irrigated conditions (referred to as drought enrichment or DE) through a variety of metagenomics sequencing techniques [[5,8–10]](https://paperpile.com/c/5tZgH3/EnmQ+z2gA+oUO7+amEd), but the degree to which this DE holds for individual root-associated *Streptomyces* remains unclear”

-Line 114-115: Please address the statistical significance of these differences. is 16.7% significantly larger than 12.3%

*We appreciate the reviewer’s attention to this point. The statistical significance for these differences is already provided in Supplementary S1 Table. In the revised version of the manuscript, we have now added the corresponding P-value as a reference in the main text to clarify this point.*

*[Line 160, Track Changes Doc] “it represented 12.3% of the total relative abundance within the roots of watered plants, and 16.7% (P=0.017, S1 Table) within drought-stressed roots.”*

-Line 175: Please add "that" between suggest and metabolite.

*[Line 222, Track Changes Doc] We have made this change.*

(Line 229-231) Why is the word "despite" the right word to use in this sentence? I have hard time seeing any contradiction between the statements in the first and second part of the sentence.

*[Line 281, Track Changes Doc] We have amended this statement and removed the word ‘despite’.*

-Line 232: Did this group include the previously used strains or were these strains completely new?

*We appreciate the reviewer’s point and have modified this statement to clarify that in this section we have a total of 48 isolates, including the original 12 and the newly added 36 strains. We would also like to note that both Fig. 5A and Fig. 6 include AC Group annotations where appropriate to visually clarify that this portion of the analysis includes the initially discussed 12 strains (which fall into the four AC Groups) and an additional 36 isolates.*

*[Line 283, Track Changes Doc] “To test this, we selected a larger cohort of 48 Streptomyces from our sorghum root-endophyte isolate collection for genomic characterization and DE phenotyping. This new cohort included all 12 isolates analyzed previously, plus an additional 36 isolates selected from available strains to maximize taxonomic breadth (based on available V3-V4 16S rRNA sequence data).”*

-Line 305-306: This has been already told in the results, and it is material that could be only in the methods.

*[Line 368-378, Track Changes Doc] We agree with the reviewer that this description of the experiment is redundant and has been removed.*

------------------------------------

**Reviewer #3:**

This manuscript clearly and logically describes an important set of studies that demonstrate the amplicon sequencing results do not necessarily connect to critical microbial functions/activities in the study system. In this case, the authors investigate well-known findings that bacteria in the genus Streptomyces increase in abundance when plants experience drought conditions. This manuscript makes important contributions to characterize which sequence variants for a V3-V4, as well as a full length 16S rRNA gene sequence to provide additional resolution. Most importantly, this study connects amplicon sequencing data to a collection of isolates to test several contributions to plant interactions, including under drought conditions. Overall, the manuscript is compelling and presents important contributions to the field. There are a couple of small clarifications and connections that could further increase the impact of these findings, particularly for the data presented in

Figures 4 and 5.

*We appreciate the Reviewer’s helpful comments on the manuscript, all of which we have attempted to address below.*

1) Figure 4 shows gene copy number for FabG and ProP. Lines 290-293 mentions 8 gene clusters annotated as FabG. Does this mean there are 8 different types of gene clusters? Do all of the genes annotated for both FabG and ProP have conserved genes surrounding it? Additional description of the gene neighborhoods for each of these genes could nicely highlight potential explanations for the results described in this paragraph.

*We agree that the terminology used here was unclear and perhaps led the Reviewer to the impression that “gene cluster” refers to a genomic locus. For clarity, we have replaced the term “gene cluster” with “orthogroup” throughout the manuscript. The annotation in question refers to groups of genes defined by homology and shared evolutionary history as called by the OrthoFinder algorithm. For example: there are 8 distinct “orthogroups” each of which was annotated with fabG (COG1028) and displayed strong association between copy number and DE.*

2) Figure 5 presents data from an important experiment and the conclusion has important implications for how the field should interpret differential abundance data in the future. However, biomass as the metric to make these conclusions is a bit removed from the actual plant physiological responses to drought. What is the impact of monoassociation on plant photosynthetic measurements, stomatal. conductance, or relative water content in leaves? Any of these measurements, particularly on interesting comparisons highlighted already by the authors (e.g. DC01, SAI126, etc.) would provide this manuscript with an important foundation for future studies.

*This is an excellent suggestion. We have available data from the original experiment that allowed us to quantify the percent water content in leaves, and have modified figure 5 (now figure 6) to include this data as a final column in our heatmap. We have added text to the Results [Line 368-402, Track Changes Doc], Discussion and Methods [Line 597, Track Changes Doc] sections of the paper in light of this new data, with additional focus on comparisons of the results for DC01 and SAI126, as suggested.*

__________________________________________________
